# Role of ventilation on the transmission of viruses in buildings, from a single zone to a multizone approach

**DOI:** 10.1111/ina.13097

**Published:** 2022-08-25

**Authors:** Gaëlle Guyot, Sabrina Sayah, Sihem Guernouti, Adeline Mélois

**Affiliations:** ^1^ Cerema BPE Research Team Nantes France; ^2^ University of Savoie Mont Blanc, CNRS, LOCIE Chambéry France

**Keywords:** aerosolized virus, airflow distribution, indoor air quality, public health, residences, virus infections, window opening

## Abstract

In a virus pandemic context, buildings ventilation has been recognized as a solution for preventing transmission of the virus in aerosolized form. The impact of the widespread recommendation of window opening and sealing door on ventilation circuits needs to be considered with a multizone approach. We modeled the airflow distribution in a building where people are isolating in a pandemic context, including one infected person. We analyzed the impact of opening the window and sealing the door in the quarantine room on exposures and probability of infection for occupants of the flat and of adjacent flats. In order to study the sensitivity of the results, we tested three ventilation systems: balanced, exhaust‐only, and humidity‐based demand‐controlled, and several window‐ and door‐opening strategies. When the door of the quarantine room is sealed, we observe that opening the window in the quarantine room always results in increased exposure and probability of infection for at least one other occupant, including in neighbors' apartments. When all internal doors are opened, we observe moderate impacts, with rather an increase of exposure of the occupants of the same apartments and of their probability of infection, and a decrease for the occupants located in other apartments. Based on the analysis on the airflows distribution in this case study, we conclude that sealing the internal door has more influence than opening the window of the quarantine room, whatever the ventilation system. We observe that this widespread recommendation to open the window of a quarantine room and to seal the door is based on the consideration of a single zone model. We illustrate the importance of moving from such a single zone approach to a multizone approach for quantifying ventilation and airing impacts in multizone buildings as residences in order to prevent epidemics of viruses such as SARS‐CoV‐2. It highlights the need of air leakage databases.

**Practical Implications**

- In an aerosolized virus pandemic context, airflow distribution in buildings is a crucial issue.
- Thus, it is important to precisely assess the airflows in the building with multizone modeling considering air leakage distributions to prevent from the virus dissemination.
- Prevention actions should secure that airflows circulate from low‐contaminated zones to high‐contaminated zones.
- The multizone modeling approach shows that opening a window and sealing the door in a high‐infected room, as a quarantine room, may cause a risk for other non‐contaminated occupants of the building.

## 1 INTRODUCTION

### 1.1 Aerosol transmission of viruses including SARS‐CoV‐2

During previous pandemics created by influenza‐type viruses (influenza, avian influenza) and corona viruses (SARS‐CoV‐1, MERS), the predominant modes of transmission were via drops, droplets, and contact with contaminated surfaces. However, evidence highlighted the risk of formation of aerosols that could travel through the air, originating from either the sneezing or the feces, depending on the environmental conditions that allow (or prevent) the survival and travel capability of the infectious aerosols.^1^, ^2^, ^3^, ^4^, ^5^

When we breathe, speak, sing, cough, and sneeze, droplets of all sizes are emitted and moved by a turbulent, hot, and humid (breathing) cloud of gas. It keeps them at a high concentration level over several meters where it transports them in a few seconds. Then, when the cloud slows down sufficiently, ventilation, specific airflow patterns become important, viral load of the emitter, duration of exposure, and susceptibility of an individual to infection are also important, as well.^3^ Using high‐frequency imaging measurements on volunteers, it has been shown that when individuals coughed and/or sneezed, in addition to the projecting drops and droplets an aerosol was formed, which could travel over distances of 6–8 m.^6^, ^7^

Aerosol transmission of SARS‐CoV‐2 was underestimated at the beginning of the pandemic. Today, several articles demonstrate that this is an important, and even the dominant, mode of transmission.^4^, ^8^, ^9^ The first experimental study by van Doremalen et al.^10^ attempts to reproduce, by nebulization, the airborne suspension of the virus after a patient had coughed. These researchers showed that SARS‐CoV‐2 could survive for 3 h as an aerosol (<5 μm). Several case studies and scientific articles continued this line of investigation and highlighted the risk of airborne transmission of the SARS‐CoV‐2.^11^, ^12^, ^13^, ^14^, ^15^, ^16^, ^17^ Several scientists acted to alert both the international scientific community and public decision‐makers. They call for aerosol transmission of the SARS‐CoV‐2 to be taken into account for more effective prevention of evolutions in the epidemic.^18^, ^19^, ^20^, ^21^, ^22^, ^23^ They reiterate the importance of taking into account more recent and accurate models of aerosol formation, phase change, and transport, which clearly call into question the current recommendations on the social distance to be maintained between people, that is, 1 to 2 m, at least indoor.^3^, ^4^

Even if various issues still need to be resolved,^24^ this way of transmission has been recognized by WHO^25^ and the American and European centers for disease prevention and control CDC,^26^, ^27^ and several countries implemented airing in the list of barrier gestures or actions on building ventilation systems, or in national protocols, in order to prevent the COVID‐19 transmission.^26^, ^28^, ^29^, ^30^

### 1.2 Key role of ventilation in buildings: energy, IAQ, and virus transmission

Ventilation and airflows in buildings constitute an important research area regarding their crucial impacts on two issues: indoor air quality and reducing heating energy consumption.

Indeed, on the contrary, since people spend 60%–90% of their life indoors (in dwellings, offices, and schools), indoor air quality (IAQ) is a major factor affecting public health.^31^, ^32^, ^33^, ^34^, ^35^ On the contrary, indoor environments can often be more polluted than outdoor ones.^36^ As a result, according to the World Health Organization,^37^ 99 000 deaths in Europe were attributable to indoor air pollution in 2012. Some authors showed that due to heavier occupation of dwellings during the COVID‐19 lockdown, coupled with changes in activities (more cooking, more cleaning) and a lack of ventilation adaptation options, indoor air pollutants could reach levels hazardous to occupants' health.^38^ They advise taking measures to address this if further stay‐at‐home orders are issued in future epidemic‐related lockdowns.

The building energy performance perspective requires efforts to rethink ventilation airflows because of their impact on thermal losses. In this context, more attention is given to the ventilation design and on‐site implementation,^39^, ^40^, ^41^, ^42^, ^43^ and ventilation systems are becoming more energy efficient with balanced systems including heat recovery or smart systems with variable airflows.^44^, ^45^, ^46^

The current COVID‐19 epidemic has highlighted another building ventilation issue: prevention of disease spread. The literature review analyzing air‐transported viruses points out a lack of data to push work forward from theory to prevention practices, particularly regarding which ventilation conditions have a strong impact on the environmental contexts that favor (or hamper) the survival and transport of infectious aerosols.^5^ Several authors have identified poor ventilation as a particular factor favoring transmission of the SARS‐CoV‐2.^3^, ^47^, ^48^ Among the best‐known examples of aerosol contamination are cases of supercontamination in places with special conditions, often in crowded and/or poorly ventilated rooms, as the Skagit Valley Choir^49^ and the Ghangzou restaurant.^11^, ^15^

Technical guidelines for building ventilation were provided by two international associations: the Federation of European of Heating, Ventilation, and Air‐conditioning Associations (REHVA), and the American Society of Heating, Refrigeration, and Air‐Conditioning Engineers (ASHRAE).^2^, ^50^, ^51^ REHVA notably raise the following points of attention concerning the ventilation of offices and buildings open to the public (excluding hospitals and health establishments):

- The need to restart ventilation at least 2 h before occupancy.
- General advice is to provide as much fresh outdoor air as is reasonably possible. The key aspect is the amount of fresh air provided per person.
- To actively use openable windows (much more than normal, even when this causes some thermal discomfort). Window opening is then the only way to boost air exchange rates. Windows should be opened for approximately 15 min when entering the room (especially when the room was occupied by others beforehand). Also, in buildings with mechanical ventilation, window opening can be used to boost ventilation further.
- Open toilet windows should be avoided to maintain negative pressure in the toilets and the right direction of mechanical ventilation.

In contrast to REHVA, ASHRAE does not recommend maximum airing but rather the minimum necessary, they warn of the risk of creating thermal discomfort which would reduce the resistance of individuals to infection. Furthermore, airing to the maximum creates energy, comfort, and indoor air quality issues.^52^, ^53^

With the exception of the last measure proposed by the REHVA (avoid opening windows in toilets), published literature and technical guidelines on ventilation rarely take into account the risk of contamination within zones of the same building. However, a few rarer published examples also demonstrate the possibility of transmission of the virus between different rooms in a building. New Zealand had set up dedicated isolation hotels where all people wishing to enter the country had to stay. Each person was regularly tested, and studies showed that aerosol contamination between rooms in this hotel was very likely.^54^ In another cluster, neighbors who had no direct contact with each other became infected in an apartment building in Seoul.^55^

To summarize, several practical recommendations on ventilation in a virus pandemic context have been published for some types of building and are very useful in the field. Nevertheless, we observe that ventilation is mainly considered from a single‐zone point of view, and not from a multizone point‐of‐view. Therefore, it is considered that the higher the air change rate (through ventilation and/or airing), the lower the risk of infection. However, we should make sure that the higher the air change rate in an area, the lower the risk of infection in that area, but also in all connected areas, which is far from obvious. We propose to address this research gap through a multi‐zone modeling approach.

### 1.3 Research issue

The novelty of this research is to better understand the interaction between airflows passing through windows, through leakage points, and through the ventilation components in multizone buildings and how they impact the effectiveness of prevention strategies, as the opening of windows and doors in a context such as the SARS‐CoV‐2 pandemic.

Multizone approach is crucial as the literature shows how CO_2_ and pollutant concentrations can strongly vary from a room to another one in residential buildings.^56^ propose to use as a metric the absolute average fractional differences—D_AAF_, defined as the difference between two concentration values (one in a room, one in another room) divided by the average of the two. It can reach 48% for VOC,^56^, ^57^ 80% for CO_2_ ^58^ to 171%^59^ for CO_2_, between the living room and one bedroom or between two bedrooms. Based on CO_2_ measurements in ten homes^60^ showed that only 60% of the homes could be considered uniform. Measured concentrations and corresponding room‐specific ventilation rates are influenced by many parameters, including the presence of a Demand‐controlled ventilation (DCV) strategy, presence of an air recirculating system, and also the position of the indoor doors,^61^, ^62^, ^63^, ^64^ none of which are influenced by the emission issue. As a result, several authors demonstrated the discrepancy between single‐zone and multi‐zone IAQ and airflow modeling in residential buildings. With a single‐zone model and a variable emission source, errors on peak concentration could be ±35% compared with a two‐zone model.^65^ With a constant emission source, errors are in a larger range (−19%; +60%).^66^ characterized air change rates and interzonal airflows in 126 residences and evaluated their effects on IAQ. Then, a two‐zone model (the bedroom and the rest of the house) calibrated with the field study was conducted for the IAQ study. They showed that 26 ± 20% of the air entering the living room comes from the bedrooms. In the IAQ modeling study, for strong sources in the bedroom, the concentrations are 65%–74% higher than those in the living room. A sensitivity analysis using the two‐zone model demonstrated that the key factors influencing pollutant concentrations are the emission source strength and location, the air change rates, and the inter‐zonal air flows. They concluded that single zone models should apply only in case of uniform emission sources.

In many buildings of Western Europe, as residences, schools, and office workplaces. whole‐house ventilation is often designed using a multizone approach, with airflows circulating between rooms.^67^, ^68^, ^69^, ^70^, ^71^ Such ventilation systems are categorized in three categories: balanced ventilation, exhaust‐only ventilation, and supply‐only ventilation. They all work on the same principle: ventilate from the less polluted zones, to the most polluted ones, generally the humid rooms: toilets, bathrooms, and kitchens.

**FIGURE 1:**
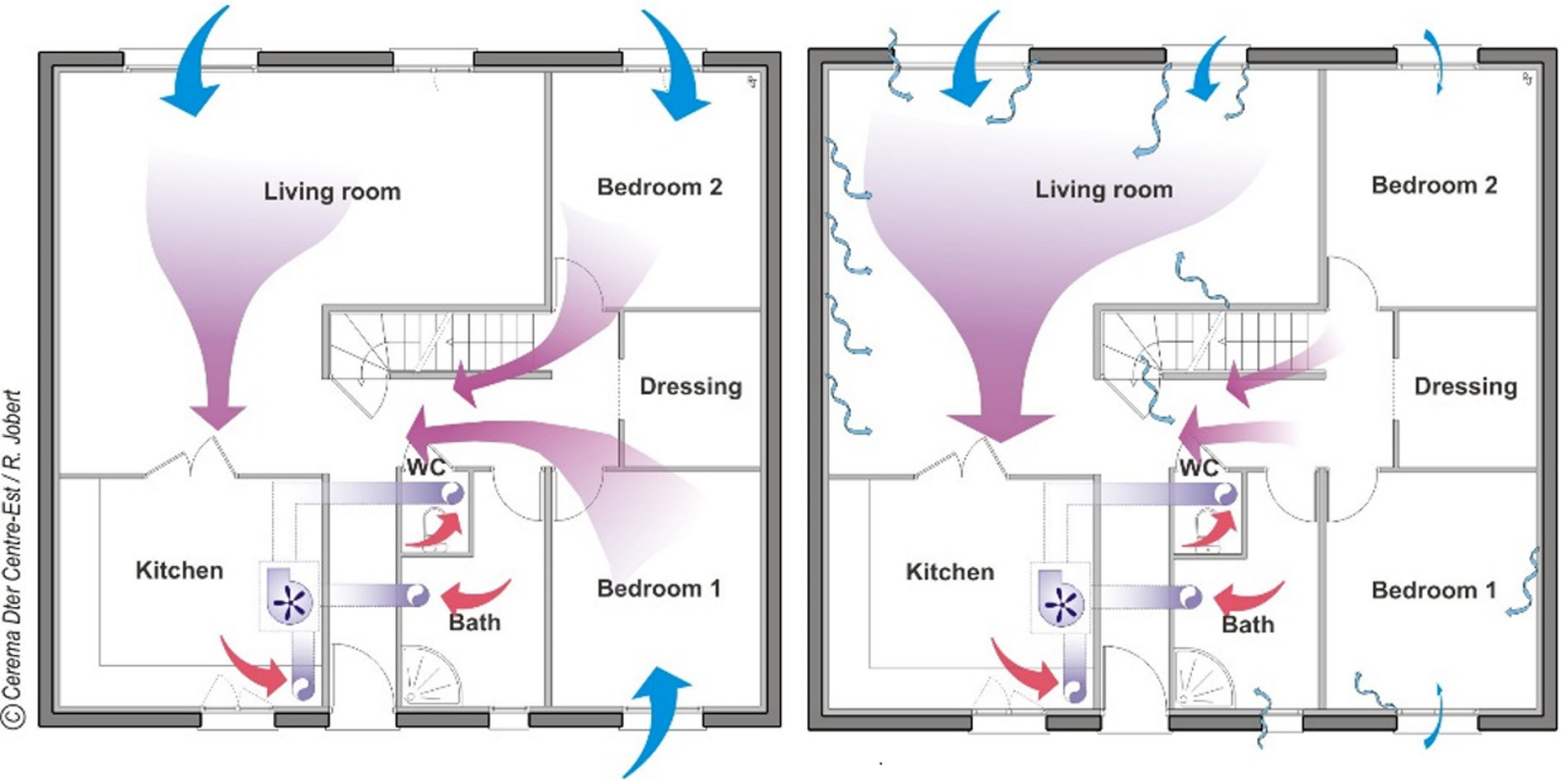
(A) Theoretical ventilation airflows in a dwelling and (B) Bypassing due to unevenly distributed air leakage in the envelope. Source: 107.

Theoretically, exhaust‐only mechanical ventilation in dwellings works on the principle of inlets drawing outdoor air into bedrooms and living rooms, and outlets exhausting air from rooms with high moisture levels (Figure 1A). Nevertheless, high air leakage on exterior walls of the higher‐humidity rooms could bypass the bedrooms, leading them to become under‐ventilated (bedrooms in Figure 1B). Opening windows amplifies this disturbance of the theoretical aeraulic circuits.

The pandemic context raises two crucial issues:

- Firstly, what is the impact of moving from a design based on air flows from the least polluted areas (bedrooms, living rooms, offices, and classrooms) to the most polluted areas (bathrooms, toilets, and kitchen), if a normally low‐pollution area becomes a high‐pollution area (e.g., a bedroom becoming a quarantine room)?
- Secondly, what is the impact of the widespread recommendation to open windows on ventilation systems, and more generally on the infection risk of occupants?

In order to answer these two questions, we model in this study the airflow distribution due to the opening of windows in a multi‐family building, where a sick person is isolated in a pandemic context such as COVID‐19. The transport of an aerosolized virus is simplified by modeling the movement of contaminated particles.

We will focus on the airflows between rooms in dwellings with high concentrations of viral particles, which will allow us to assess the exposure of occupants to the contaminated aerosol and the risk of infection.

To investigate the sensitivity of the results, we test three ventilation systems, a balanced constant airflow system, a constant airflow system with exhaust only, a humidity‐controlled demand system, and several strategies for opening windows and doors to maximize the air change rate in the rooms.

## 2 METHODS

### 2.1 Presentation of the case study

**FIGURE 2:**
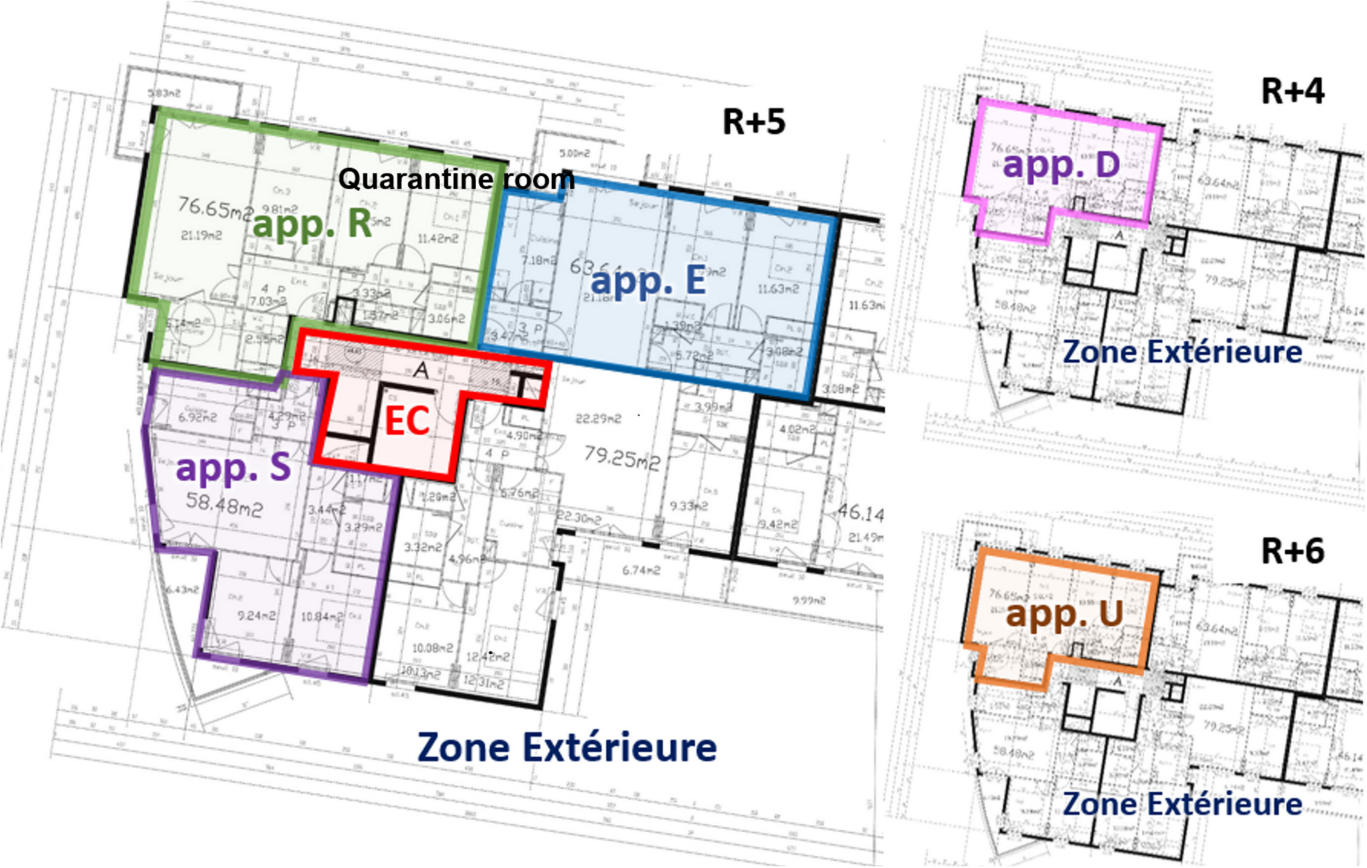
Plan of the studied multi‐family building: the reference apartment (App. R) is at the 5th level (R + 5), below the app. U and above the app. D.

The case study is a real multi‐family building with houses rental accommodation, spread over eight floors. We are particularly interested in one of the flats in the building, called the “reference apartment” (or app. R). It is a 3‐BR flat on the fifth floor. Its neighborhood consists of four other flats arranged as in Figure 2: two identical flats below (app. D) and above (app. U), as well as two flats on the same floor, a 3‐BR flat to the south (app. S), and a 3‐BR flat to the east (app. E). To the southwest are the staircase (CE) of the building. The modeling of the staircase is highly simplified. It is modeled as a transfer zone, unventilated, heated, with no leakage to the outside. Indeed, we focus in this approach on direct transfer between flats, and therefore underestimate the risk of transmission.

**FIGURE 3:**
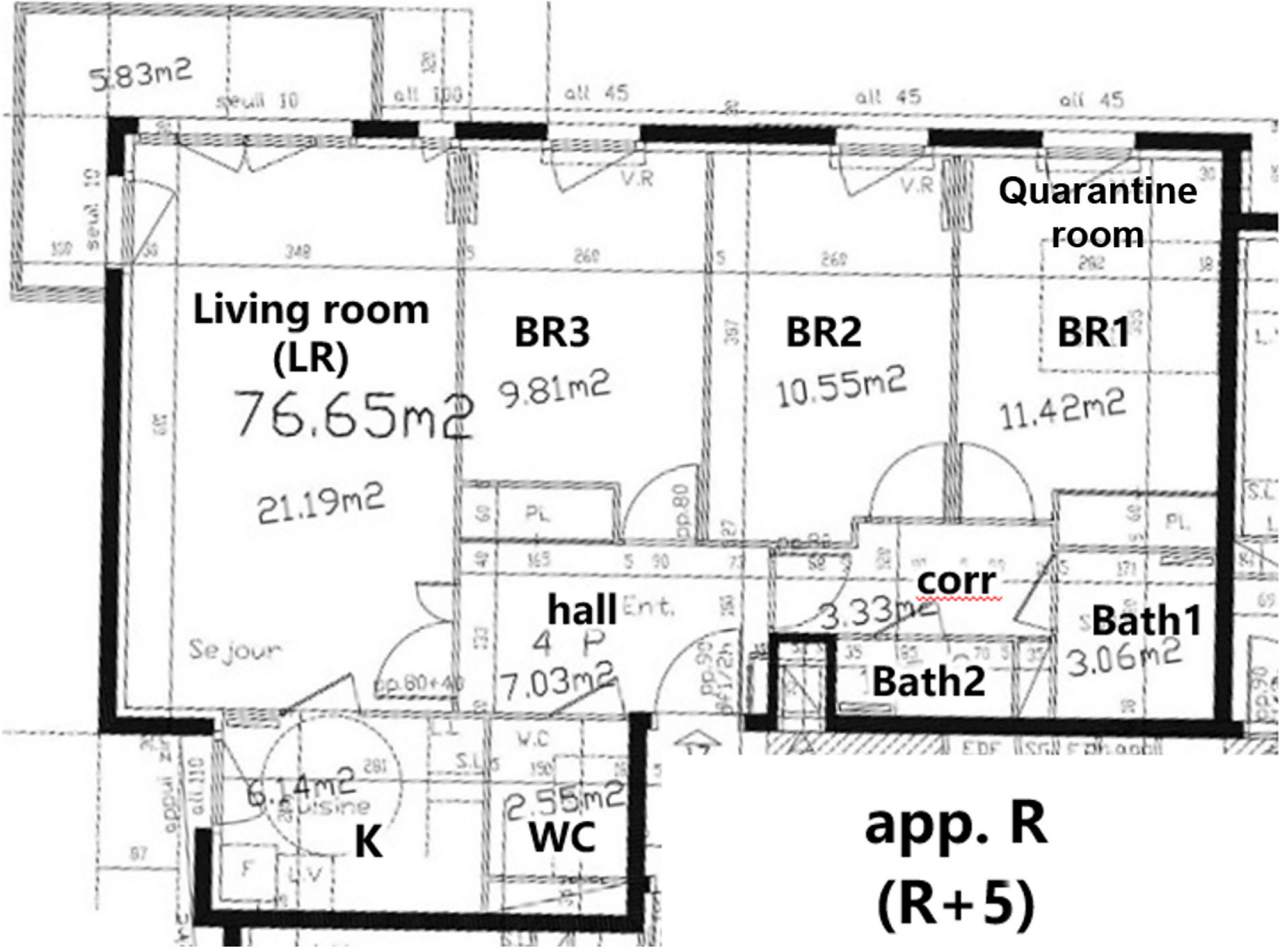
Plan of the reference apartment studied.

The reference apartment is divided into 10 rooms, see Figure 3: a living room (LR), a separate kitchen (K), three bedrooms (BR1, BR2, BR3), an entrance hall (hall), a corridor (corr), a bathroom (Bath1), a shower room (Bath2), and a toilet. The total heated volume is 197 m^3^. Overall air leakage from the envelope has been measured applying the ISO 9972 procedure; and is *n*_50_ = 1.5 h^−1^.

We assume that two inhabitants live in the master bedroom (BR2 in the flat) and one inhabitant in each other bedroom. Thus, the 3BR‐ and 2BR‐flats have four and three occupants, respectively. All occupants are isolating in their homes in a pandemic context such as the first wave of COVID‐19 in 2020.

In the reference apartment (app. R), one of the occupants (Occ. 11) is infected and is quarantined in his bedroom, BR1. He stays in this room all day long, except for 40 min in the bathroom (Bath1) in the evening.

For the time spent in the various rooms by the other occupants, we use detailed data from the French national campaign on the IAQ of dwellings from 2005,^34^ based on a representative sample of the population and including 567 dwellings and 1612 occupants. According to these data, each day people spend on average around 2 h 40 min in the kitchen, 2 h 50 min in the living room, and 40 min in bathrooms. In the particular context of lockdown, we suppose that the rest of the time is spent in the bedrooms, with people either working, studying, or sleeping. Thus, for each dwelling, most occupants stay alone in the room in which they sleep at night, and one occupant of the master bedroom moves into the kitchen to be alone. The selected occupancy schedules for the occupants are presented in Table 2. We focus our analysis on the most exposed occupants, in bold in this table.

### 2.2 Modeling

#### 2.2.1 Multizone approach

We investigate airflows and particle concentrations using numerical modeling with CONTAM software.^72^, ^73^, ^74^ The validation of such multizone models is well documented and has been compared with experimental results.^74^, ^75^, ^76^, ^77^, ^78^, ^79^ Several authors have shown that this type of model, assuming well‐mixed air in every zone, is a good fit for ventilation and IAQ modeling.^80^, ^81^ Each room in the house is modeled as one air zone (making a total of 11 zones). The indoor temperature is assumed to be maintained at 20°C during this period, by means of the heating system.^82^

The air mass balance equation is considered for each zone as follows:

(1) $$\sum_{j}q_{i,j}+q_{V,i}=0$$

Where $q_{i,j}$ are the mass airflows entering or leaving zone *i* through leaks, door undercuts, and passive components (trickle vents/grilles on external walls) from all the adjacent zones *j* (and outside), *q*_*V,I*_ is the airflow extracted or supplied by the mechanical ventilation system in zone *i*.

Airflow rate through leaks is calculated from the pressures and the leakage characteristics, using the power law (Equation 2).

(2) $$q_{i,j}=\rho_{i}C_{i,j}∆P_{i,j}^{n}\;$$

Where $\rho_{i}$ is the air density in the origin zone i [kg m^−3^], $C_{i,j}$ the air leakage coefficient [m^3^ s^−1^ Pa^−n^], $∆P_{i,j}$ the pressure difference between zones *i* and *j* [Pa], and n the flow exponent [−].

The zones' pressures depend on differences in air density (stack effect, based on the hydrostatic equation). The equation requires input of the pressure on façades between the zones and outside, which is linked to the wind regime as follows:

(3) $$P_{v,i}=\frac{1}{2}\rho_{\text{out}}c_{P,i}v_{\text{build}}^{2}$$

Where $P_{v,i}$ is the wind pressure on the outdoor façade in question, that of zone *i*, $\rho_{\text{out}}$ is the density of outside air, $c_{P,i}$ is the pressure coefficient at the leak point [−], and $v_{\text{build}}$ is the wind velocity at the building height [m s^−1^].

Once the airflow rates are known, the concentrations of pollutants are obtained by calculating (a) the pollutant transport due to airflows and (b) the mass balance for each zone.

#### 2.2.2 Weather data

We use typical dynamic meteorological data for two winter weeks in Paris, France, on a 10‐min time step (ASHRAE IWEC Weather file, 2001, Table 1), from 00:00 a.m. on January 1st until 00:00 a.m. on January 16th, accounting for 360 simulated hours, in order to simulate winter weather conditions during a 2‐week stay‐at‐home order.

**TABLE 1:** Overview of the dynamic weather data parameters for the selected winter weeks for Paris

	Temperature (K)	Pressure (Pa)	Wind velocity (m s^−1^)	Wind direction (°)	Humidity ratio (g kg^−1^)
Median value	283.3	99 500	3.4	140	6.0
1st quartile	279.75	98 900	2.1	120	5.1
3rd quartile	286.6	100 500	4.4	180	6.5

**TABLE 2:** Occupancy schedules selected for the study

Occupant nb	Apartment	In living‐room	In kitchen	In bathroom	In bedroom
1	App_U	6 h35–8 h30 12 h30–14 h00 19 h00–20 h20	6 h20–6 h35 12 h00–12 h30 19 h00–19 h40	Bathroom2 20 h20–21 h00	BR3 8 h30–12 h00 14 h00–19 h00 21 h–6 h20
2	App U	7 h00–8 h30 12 h30–14 h00 19 h40–21 h00	8 h30–12 h30 14 h00–19 h40	Bathroom1 6 h20–7 h00	BR2 21 h00–6 h20
3	App U	6 h35–7 h00 7 h40–8 h30 19 h40–21 h00	6 h20–6 h35 12 h00–12 h30 19 h00–19 h40	Bathroom1 7 h00–7 h40	BR2 8 h30–12 h00 12 h30–14 h00 21 h00–6 h20
**4**	**App U**	**6 h35**–**8 h30** **12 h30**–**14 h00** **19 h00**–**20 h20**	**6 h20**–**6 h35** **12 h00**–**12 h30** **19 h00**–**19 h40**	**Bathroom1** **20 h20**–**21 h00**	**BR1** **21 h**–**6 h20**
5	App E	6 h35–8 h30 12 h30–14 h00 19 h00–20 h20	6 h20–6 h35 12 h00–12 h30 19 h00–19 h40	Bathroom 20 h20–21 h00	BR2 21 h–6 h20
**6**	**App E**	**7 h00**–**8 h30** **12 h30**–**14 h00** **19 h40**–**21 h00**	**8 h30**–**12 h30** **14 h00 ‐19 h40**	**Bathroom** **6 h20**–**7 h00**	**BR1** **21 h00**–**6 h20**
7	App E	6 h35–7 h00 7 h40–8 h30 19 h40–21 h00	6 h20–6 h35 12 h00–12 h30	Bathroom 7 h00–7 h40	BR1 8 h30–12 h00 12 h30–14 h00 21 h00–6 h20
**8**	**App R**	**6 h35**–**8 h30** **12 h30**–**14 h00** **19 h00**–**20 h20**	**6 h20**–**6 h35** **12 h00**–**12 h30** **19 h00**–**19 h40**	**Bathroom2** **20 h20**–**21 h00**	**BR3** **8 h30**–**12 h00** **14 h00**–**19 h00** **21 h**–**6 h20**
**9**	**App R**	**7 h00**–**8 h30** **12 h30**–**14 h00** **19 h40**–**21 h00**	**8 h30**–**12 h30** **14 h00**–**19 h40**	**Bathroom1** **6 h20**–**7 h00**	**BR2** **21 h00**–**6 h20**
**10**	**App R**	**6 h35**–**7 h00** **7 h40**–**8 h30** **19 h40**–**21 h00**	**6 h20**–**6 h35** **12 h00**–**12 h30** **19 h00**–**19 h40**	**Bathroom1** **7 h00**–**7 h40**	**BR2** **8 h30**–**12 h00** **12 h30**–**14 h00** **21 h00**–**6 h20**
**11**	**App _R**			**WC** **20 h20**–**21 h00**	**BR1** **21 h00 ‐20 h20**
12	App S	6 h35–8 h30 12 h30–14 h00 19 h00–20 h20	6 h20–6 h35 12 h00–12 h30 19 h00–19 h40	Bathroom 20 h20–21 h00	BR2 21 h–6 h20
13	App S	7 h00–8 h30 12 h30–14 h00 19 h40–21 h00	8 h30–12 h30 14 h00 ‐19 h40	Bathroom 6 h20–7 h00	BR1 21 h00–6 h20
14	App S	6 h35–7 h00 7 h40–8 h30 19 h40–21 h00	6 h20–6 h35 12 h00–12 h30 19 h00–19 h40	Bathroom 7 h00–7 h40	BR1 8 h30–12 h00 12 h30–14 h00 21 h00–6 h20
15	App D	6 h35–8 h30 12 h30–14 h00 19 h00–20 h20	6 h20–6 h35 12 h00–12 h30 19 h00–19 h40	Bathroom2 20 h20–21 h00	BR3 8 h30–12 h00 14 h00–19 h00 21 h00–6 h20
16	App D	7 h00–8 h30 12 h30–14 h00 19 h40–21 h00	8 h30–12 h30 14 h00 ‐19 h40	Bathroom1 6 h20–7 h00	BR2 21 h00–6 h20
17	App D	6 h35–7 h00 7 h40–8 h30 19 h40–21 h00	6 h20–6 h35 12 h00–12 h30	Bathroom1 7 h00–7 h40	BR2 8 h30–12 h00 12 h30–14 h00 21 h00–6 h20
**18**	**App D**	**6 h35**–**8 h30** **12 h30**–**14 h00** **19 h00**–**20 h20**	**6 h20**–**6 h35** **12 h00**–**12 h30** **19 h00**–**19 h40**	**Bathroom1** **20 h20**–**21 h00**	**BR1** **21 h**–**6 h20**

*Note*: In bold, the most exposed occupants.

**TABLE 3:** Extracted airflows for EV and BV systems

Case study	Zone	Q (m^3^ h^−1^)	Qpeak (m^3^ h^−1^) ‐ 12:00–13:00
			19:00–20:00
Single‐family house (4BR)	Kitchen	45	135
	WC 1 & 2	15	
	Bathroom 1 &2	30	
App R (3BR) App D (3BR) App U (3BR)	Kitchen	45	120
	WC	30	
	Bathroom 1 &2	30	
App E (2BR) App S (2BR)	Kitchen	45	105
	WC	15	
	Bathroom	30	

**TABLE 4:** Supplied airflows for BV systems

Case study	Zone	Q (m^3^ h^−1^)	Qpeak (m^3^ h^−1^) ‐ 12:00–13:00
			19:00–20:00
Single‐family house (4BR)	Living room	36.6	64.4
	Bedrooms	19.3	32.2
App R (3BR) App D (3BR) App U (3BR)	Living room	54	84
	Bedrooms	27	42
App E (2BR) App S (2BR)	Living room	45	75
	Bedrooms	22.5	37.5

**TABLE 5:** Definition of the studied mitigation scenarios based on different window‐ and internal door‐opening scenarios and assessment strategies

	Quarantine room window	Quarantine room door	Other internal doors	3 other windows ‐same facade	3 other windows ‐opposite facade
*Reference case*	*Closed*	*Closed*	*Closed*	*Closed*	*Closed*
Scenario 1‐HCPB	Open	Sealed	Closed	Closed	Closed
Scenario 2‐ low diluting strategy	Open	Open	Closed	Closed	Closed
Scenario 3‐ high diluting strategy	Open	Open	Open	Closed	Closed
Scenario 4 ‐ balanced strategy	Open	Sealed	Closed	Open	Closed
Scenario 5 ‐ balanced strategy	Open	Sealed	Closed	Closed	Open
Scenario 6 ‐ entering airflow limiting strategy	Half‐open	Sealed	Closed	Closed	Closed

**TABLE 6:** Relative exposures of the occupants for the 6 studied scenarios, with exhaust‐only ventilation (EV)

	1‐Window	2‐Window+door	3‐Window+alldoors	4‐3windows	5‐3windows‐sameside	6‐Window‐halfopen
Occ. 4 (Appt U)	286%	−61%	−58%	286%	286%	328%
Occ. 6 (Appt E)	155%	−44%	−46%	155%	155%	212%
Occ. 8 (Appt R)	−82%	8%	26%	−84%	−84%	−77%
Occ 9 (Appt R)	377%	−8%	3%	376%	376%	393%
Occ. 10 (Appt R)	574%	−22%	−16%	527%	527%	644%
Occ. 11 (Appt R‐quarantine)	46%	−20%	−18%	46%	46%	64%
Occ. 18 (Appt D)	252 350%	451%	683%	252 454%	252 458%	280 450%

**TABLE 7:** Infection risk by occupant (%) for the 6 studied scenarios, with exhaust‐only ventilation (EV)

	1‐Window	2‐Window + door	3‐Window + alldoors	4‐3windows	5‐3windows‐sameside	6‐Window‐halfopen
Occ. 4 (Appt U)	5.4%	0.5%	0.6%	5.4%	5.4%	6.0%
Occ. 6 (Appt E)	0.7%	0.2%	0.1%	0.7%	0.7%	0.9%
Occ. 8 (Appt R)	0.1%	0.8%	1.0%	0.1%	0.1%	0.2%
Occ 9 (Appt R)	6.4%	1.3%	1.4%	6.4%	6.4%	6.6%
Occ. 10 (Appt R)	10.7%	1.3%	1.4%	10.0%	10.0%	11.7%
Occ. 18 (Appt D)	3.6%	0.0%	0.0%	3.6%	3.6%	4.0%

**TABLE 8:** Cumulated airflows (kg s^−1^) through the different walls of the quarantine room for the 6 studied scenarios, with exhaust‐only ventilation (EV)

	1‐Window	2‐Window + door	3‐Window + alldoors	4‐3windows	5‐3windows‐sameside	6‐Window‐halfopen	7‐Reference
Ext‐inf	1.9	6.9	6.9	1.9	1.9	1.9	6.3
BR2‐inf	−1.8	0.6	0.0	−1.7	−1.7	‐1.8	−0.1
Corr‐inf	−0.2	0.0	0.0	−0.2	−0.2	−0.2	−0.1
Bath1‐inf	−1.1	−0.4	0.0	−1.1	−1.1	−1.1	−0.6
Kitch‐inf (Appt E)	−0.4	−0.2	−0.2	−0.4	−0.4	−0.4	−0.2
Floor‐inf (Appt D)	−0.7	0.5	0.5	−0.7	−0.7	−0.7	0.3
Ceiling‐inf (Appt U)	−1.0	−0.1	−0.1	−1.0	−1.0	−1.0	−0.3
Door	0.0	−19.1	−19.9	0.0	0.0	0.0	−13.6
Window	16.6	15.3	14.8	16.7	16.7	2.0	0.0
Trickle vent	3.1	8.8	8.8	3.1	3.1	3.1	8.3

The wind velocity, retrieved from weather data acquired at a height of 10 m and in an open area, is recalculated for the particular site; this is done using a power law with factors specific to a suburban area, and for the house height, 8.5 m. The pressure coefficients from standard EN 15242 are used, assuming no barriers, that is, +0.5 on the upwind façades and −0.7 on the downwind façades.

#### 2.2.3 Air leakage distribution

In practice, leaks are everywhere in buildings (around windows, electric components, and cracks).^83^ In order to simplify this complex distribution, we modeled air leakage in CONTAM by one path at the center of each external and internal partition wall in each zone, using the power law (Equation 2).

For the external walls of the multi‐family building, we use data from on‐site measurements as the permeability of each dwelling was measured. For the apartments R,D,U,E,S, it was measured, respectively, as *n*_50_ = 1.5–0.7–1.8–1.4–2.3 h^−1^. We consider an even distribution on the prorata of the external wall surfaces. For the internal partition walls of the same dwellings, we take the value of *q*_50_ proposed by^84^ for heavy structures: 1.2 m^3^ h^−1^ m^−2^.

For leakage between the walls separating the different dwellings of the building, we found little data in the literature, except four studies. Bohac et al.^85^ studied six multi‐family residential buildings in the United States, for which median airflow values from adjacent flats were characterized in the range [2%–35%] of the total airflow in the unit, and the median internal air leakage to the adjacent flats accounted for 27% of the total air leakage from the unit. Air leakage between flats in multi‐family residential buildings was reported to account for 12%–33% of total air leakage at 50 Pa in a Swedish study.^86^ In an experimental study conducted on six flats in four multi‐family residential buildings located in Quebec, Canada, the authors measured internal air leakage of 19%–22%–34%–64%–65%–67% of the total air leakage of the six flats.^87^ Specifically, they characterized the proportion between adjacent flats and common areas/halls. The latter walls are more airtight, accounting for 11%‐n/a‐32%–46%–37%–52% of the total air leakage from the flat, respectively. A final study proposes values to be provided to the model for leakage rates at 50 Pa pressure (*q*_50_) for each type of wall separating two parts of a multi‐family building. These are the data from the study by Lozinsky and Touchie,^88^ which is based on recent inter‐zonal air leakage tests conducted in 12 newly constructed multi‐family buildings in Canada. In conclusion, after having analyzed all these data from the literature, the data that we use in our modeling are calculated from the proportions of *q*_50_ proposed by the last study^88^ and the value of the overall measured air leakage of the building. Thus, the values of the model are as follows: *q*_50_ = 0.504 m^3^ h^−1^ m^−2^ for ceilings and floors; *q*_50_ = 0.07 m^3^ h^−1^ m^−2^ for vertical walls between two neighboring flats; and *q*_50_ = 4.01 m^3^ h^−1^ m^−2^ for walls facing the common corridor of the building. It should be noted that these values are quite low, so our study tends to minimize virus circulation through permeability defects between dwellings.

#### 2.2.4 Three modeled ventilation systems

We study three options for the ventilation systems, selecting reference systems at least in France but also in Western Europe.^67^, ^68^, ^69^, ^70^, ^71^ They are whole‐house systems complying with the French airing regulation,^89^ which provides extract units in wet rooms and components to allow fresh air into living and bedrooms:

- A balanced constant airflow ventilation system (BV)
- An exhaust‐only constant airflow ventilation system (extracted airflows are the same for 1 and 2) (EV)
- A humidity‐based demand‐controlled ventilation (RH‐DCV) system.

For the BV and the EV systems, the values of the exhaust airflows in wet rooms in the model are those provided by the French regulation.^89^ Also, we assume that the extracted airflow is switched on twice a day for 1 h to a peak value during cooking periods. The extracted airflows are given in Table 3. As a result, the total exhaust airflow for the reference apartment is 105 m^3^ h^−1^, accounting for an average dwelling air change rate of 0.5 h^−1^, potentially increased to 1 h^−1^ during cooking periods.

In the BV system, each bedroom is equipped with a supply vent, while the living room has two such vents, sized to balance the total exhaust flow. Supplied airflows are given in Table 4. In the EV system, these seven air supply vents are replaced with self‐regulating trickle vents on windows, designed to be able to balance the total exhaust airflow. To model airflows through these trickle vents, we match the calculated operating curve with data from the fan manufacturer and adjust the power law (Equation 2) as shown in (Equation 4). The calculated flow exponent is consistent with the published literature.^90^

(4) $$q_{\text{out},i}=\rho_{\text{out}}4.79∆P_{\text{outdoor},i}^{0.53}$$

Where $\rho_{\text{out}}\;$is the density of outside air [kg m^−3^] and $∆P_{\text{outdoor},i}$the pressure difference on the façade in question between outside and zone *i* [Pa].

- The RH‐DCV system has received an agreement to be allowed in all new French residential buildings.^44^, ^91^, ^92^ It adjusts the airflows according to the direct relative humidity (RH) measurement, through the extensions and retractions of a hygroscopic fabric modifying the cross‐section of inlets and outlets. In our case study, this system includes:
- A kitchen exhaust providing an airflow between 15 and 55 m^3^ h^−1^, and a peak airflow of 135 m^3^ h^−1^ for 30 min if activated by the user,
- Bathrooms exhaust providing an airflow between 5 and 45 m^3^ h^−1^,
- Toilets exhaust providing a constant airflow of 5 m^3^ h^−1^, which could be switched to 30 m^3^ h^−1^ for 20 min thanks to an occupancy sensor,
- A trickle vent in every bedroom and two in the living room, with an operating rate between 4 m^3^ h^−1^ and 31 m^3^ h^−1^ (reference pressure of 10 Pa).

#### 2.2.5 Door and window opening ‐ modeling and scenarios

This study considers a situation in which different windows are opened for 15 min, three times a day. Airflows through the open windows are modeled using a two‐way flow model, with a neutral height (air can enter and exit at the same time), and a discharge coefficient of 0.78 as proposed by Weber and Kearney,^93^ for an opening measuring 1.2 m × 1.35 m.

For half‐open windows, we use the same model but with a height of 0.3 m instead of 1.2 m. For open internal doors, airflows are calculated using the same two‐way flow model as for open windows. In other cases, internal doors are assumed to be closed, as observed in the campaign carried out by Bernard.^94^ In this case, a door undercut is generally required by the ventilation regulations and can be modeled through a single 1‐cm‐high crack. For sealed doors, no undercut is modeled.

#### 2.2.6 Contaminated particles—emission and transfer

There are still many uncertainties about the characterization of the SARS‐CoV‐2 aerosols, their reactivity on surfaces and the size of the agglomerated particles that may contain the SARS‐CoV‐2; in any case, these may vary in size depending on the situation. The virus particle has a diameter of 80–160 nm but could agglomerate with other particles to reach sizes of up to 5 μm.^2^ The aerosolized virus is considered a pollutant, in precisely the same way as contaminated particles transferred through the air. We ignore reactions with other indoor air pollutants in particle form, despite the fact that the levels of these are known to be high in residential buildings.

Studies show that the rate of virus release depends strongly on the activity of the infected person. Uncertainties remain about the exact values, but Buonnano^95^ proposes values depending on two cross‐cutting factors: the occupant's physical activity (resting, standing, light exercise, and intense exercise), and the occupant's oral activity (breathing, talking, or loud talking). These values range from 2 quanta h^−1^ for a person who is silent at rest to 408 quanta h^−1^ for a person who is speaking loudly while doing intense exercise.

In our study, as in the calculation tool developed by REHVA,^96^ we assume that the infected occupant is at rest throughout the modeling, emitting contaminated particles at a constant rate, which corresponds to the 90^ème^ percentile of Buonnano^97^: 3.1 quanta h^−1^. We also introduce the decay rate of the virus λ, its value varies between 0 and 0.63 h^−1^. We select the average of the literature values: λ = 0.32 h^−1.^ ^49^, ^96^ Then, we calculate the virus quantity according to (Equation 5).

(5) $$S_{\mathrm{cov}}\left(t\right)=G_{\mathrm{cov}}e^{-t/t_{c}}$$

With *S*_cov_ virus quantity, *G*_cov_ the initial emission rate, and *t*_*c*_ the decay time constant, corresponding to 1/*λ*.

In addition, the deposition phenomenon of the virus is taken into account, depending on the deposition rate of the particles and the volume of the room in question. The literature shows that the deposition rate of SARS‐CoV‐2 can vary between 0.24 and 1.5 h^−1^ depending on the particle size, the value 0.3 h^−1^ is assumed.^96^ Thus, in each zone of the building modeled on CONTAM, a “sink” type element representing the deposition phenomenon is introduced. It removes the virus particles according to the following equation:

(6) $$R_{\mathrm{cov}}\left(t\right)=k_{d}\;V_{z}\;\rho_{\mathrm{air}}\left(t\right)\;C_{\mathrm{cov}}\left(t\right)$$

Where *R*_cov_(*t*) is the removal rate (by deposition) [quanta s^−1^], *V*_*z*_ is the volume of the zone [m^3^], *k*_*d*_ is the deposition rate [1/s], *ρ*_air_(*t*) is the air density [kg_air_ m^−3^], and *C*_cov_ (*t*) is the concentration of aerosolized virus [quanta kg_air_^−1^].

After being shed by the infected occupant, these contaminated particles in the air move around within the different rooms of the house, due to the pressure differences between zones. They are carried in air through leak points in external and internal partition walls, through door undercuts if doors are closed, through windows if these are open, and through ventilation components (air inlets and outlets).

As regards passage through leak points, we need to consider the penetration factor; the higher this factor, the more the particles can penetrate via the leak points. A penetration factor of one means that the leak point presents no obstacle to the passage of the particle.

Liu and Nazaroff^98^ measured penetration factors in the laboratory, for different particle sizes (0.02–7 μm) and for cracks with variable geometry (height from 0.25 to 1 mm, length from 4 to 10 cm), on various materials (aluminum, brick, concrete, timber, and plywood), with a pressure reduction of 4 or 10 Pascals. They then compared their measurements with results from the model they developed. They conclude that for particles with a diameter of 0.1–1.0 μm, with a depression >4 Pascals, this penetration factor is close to 1 (no filtration). They also showed that on an aluminum plate, with a depression of 4 Pascals, the penetration factor is lowered to 0.2 for particles of diameter >1 μm passing through a crack of height 0.25 mm and length 9.4 mm, or for particles of diameter >2 μm for a crack of the same height and length 4.3 cm. If the height of the crack increases and is 1 mm, then, the penetration factor is much higher, and is close to 1. The authors also stressed the importance of the deposition of particles of size 0.1–0.4 mm on the surface of the leaks, which then decreases the penetration factor.

The literature review by Chen and Zhao^99^ proposes values for penetration factors through different airtightness defects measured in the laboratory and in real buildings. The penetration factors measured in real buildings range from 0.6 to 1.0 for particles with diameters of 0.05 and 2 μm. For larger particles, the penetration factors decrease as the gravitational effects increase. For smaller particles, the penetration factors are also lower due to Brownian diffusion effects.

With these data from the literature and facing many uncertainties about characterization of the SARS‐CoV‐2 aerosols, we assume a penetration factor of 1. Indeed, once a window is opened, the pressures exerted on the interior walls can reach levels similar to the pressures normally exerted on exterior walls, regularly reaching −2 Pa, and as low as −7 to −11 Pa (strong wind) in our simulations.

### 2.3 Reference case and studied scenarios

In the reference case, all the internal doors and all the windows of the house are closed. We will also follow the recommendation of the French high council for public health (HCSP)^28^ of opening the window and seal the door of the quarantine room, in all our scenarios, with windows being opened three times a day for 15 min at the following times: at 8:00 a.m., at 12:00 p.m., and at 6:00 p.m. The neighboring flats all have, in all scenarios, all doors and windows closed.

To study the sensitivity of our results, we studied six mitigation scenarios, summarized in Table 5.

- Scenario 1 corresponds exactly to the strategy recommended by the French high council for public health,^28^
- Scenario 2 corresponds to a virus‐diluting strategy with the corridor, in order to avoid an excessive concentration in the quarantine room which could enable cross‐contamination with the connected rooms (room above, room below, or adjacent rooms), where the other occupants spend most of their time,
- Scenario 3 corresponds to a strategy of further diluting the virus concentration, with all the doors between rooms open. Diluting strategies could be more useful later, once we know the contaminating dose of the virus,
- Scenarios 4 and 5 are used to assess the effect of opening, at the same time, three other windows in the dwelling, in order to balance the contaminating airflows from the quarantine room to the other zones. Windows are opened on the same building façade as the quarantine room (Scenario 5) or on the same façade and also the opposite one (Scenario 4),
- Scenario 6 assesses the effect of only half‐opening the windows of the quarantine room, in order to reduce the airflow entering via this route, which according to the mass balance approach is supposed to be transferred to other connected rooms.

### 2.4 Relative exposure and probability of infection as performance indicators

As a very common indicator by^100^, ^101^, ^102^, ^103^, ^104^ and as proposed by,^3^ in this study, we firstly examine the cumulative exposure to the contaminated particles Ej, for each occupant j (Equation 7). We calculate it, and we compare it to the reference case exposure, in order to assess the relative performances of different strategies.

(7) $$E_{j}=\sum_{i}C_{j}\left(t_{i}\right)*∆t_{i}$$

where *C*_*j*_ (*t*_*i*_) is the exposure concentration for occupant *j* at the time step $∆t_{i}=10\;\min$.Since the infecting dose necessary for SARS‐CoV‐2 to transmit successfully is still unknown, as for other viruses, our results are valuable only for the purposes of making a relative comparison, and not for their absolute values.

This cumulative exposure is calculated considering the occupancy schedules given in Table 1, over the 2‐week simulated period (360 h). This is done using the PYTHON package for analysis of the CONTAM output data, which accounts for concentrations in the 12 zones for each of the 2160 time steps.

Moreover, many studies of virus spread with ventilation use the Wells Riley equation to calculate the probability of infection. Most of these studies are single‐zone models that focus on the room in which the infected person is located, so a simplified equation take into account a single concentration and ventilation rate. To study the phenomena at the scale of a building, it is necessary to go back to the source equation for a multi‐zone analysis, as in the study by Pease.^105^ The probability of infection P is calculated, based on a Poisson distribution, as follows:

(8) $$P=1-e^{-\mu }$$

where *μ* is the average number of quanta (one quantum gives a 63% probability of inducing infection) breathed in by a person likely to be infected, that is, a person who is exposed to the virus.

To obtain the average number of quanta breathed from the concentration of quanta, we use the method of Rudnich and Milton^105^, ^106^:

(9) $$\mu =p\int_{t1}^{t2}C_{i}\mathrm{dt}$$

where *C*_*i*_ are the concentrations in quanta per volume, *p* is the volumetric breathing rate, and *t*_1_ and *t*_2_ are the start and end times of exposure (*t*_2_ > *t*_1_ ≥ 0).

With the CONTAM simulation results, the values of the particulate matter concentrations in the room where the occupant is located for each time step can be collected for each occupant. The concentrations for each occupant are summed over a day and then multiplied by the volumetric breathing rate *p* = 0.48 m^3^ h^−1^, to obtain the value of the average number of quanta breathed, then the probability of infection *p* is deduced.

## 3 RESULTS AND DISCUSSION

The results include the occupants' exposure for different strategies of windows and doors opening, presented in comparison with the reference case (windows and doors), the probability of infection of the occupants, and the airflows through the walls of the quarantine room. We present in detail the EV case, probably the most common case in the Western European residential building stock.^67^, ^68^, ^69^, ^70^, ^71^ Then, we give an overview of the results with the two other ventilation systems.

### 3.1 Exhaust‐only ventilation (EV)

The relative exposures of the seven most exposed occupants of the multi‐family building (in the reference flat and in the adjacent flats) compared with the reference case, for a building equipped with EV are given in Table 6. As this type of ventilation system extracts airflows from high‐moisture rooms, it creates a pressure difference with respect to the living room and bedrooms, where fresh air enters through grilles placed in the wall or on the windows, thanks to the pressure differences created, plus the wind and stack effects. With this kind of ventilation system, there are higher pressure differences between rooms (than with balanced ones), which causes air to flow from room to room. In this case, we observe that opening the window in the quarantine room always results in increased exposure for at least one other occupant, including in neighbors' dwellings. Some scenarios even cause extremely high relative increases. Indeed, the scenarios can be separated into two groups: the scenarios where the door of the quarantine room is sealed (sc.1,4,5,&6) and the scenarios with dilution strategies (sc. 2&3) where this door is open. The first group shows extreme increases in relative exposures compared with the reference case, while the second group shows moderate increases.

More precisely, we observe that:

- The different scenarios are not all beneficial even for the quarantined occupant (Occ. 11). The exposure of the contaminated occupant increases by 46% for an open window in the quarantine room, and by 64% for a semi‐open window. In the case of dilution strategies (sc. 2&3), the exposure of this occupant decreases by −18% to −20%.
- In the first group of scenarios, the three occupants living in adjacent apartments experience an extreme increase in exposure: +155% (Occ. 6, same floor); +286% (Occ 4, floor above); +2.8 10^5^% (Occ 18, floor below). This last extreme variation reflects a very low absolute value in the reference case. In addition, inside the reference apartment, two occupants exposures increase (+377% and + 574%) and one decrease (−82%).
- The dilution strategies show much better results. Indeed, the exposure rates decrease for four occupants in the high dilution scenario (sc.3), from −16% to −58%, and for 3 occupants in the low dilution scenario (sc.2), from −20% to −61%. The low dilution scenario is the most effective since only one occupant of the reference apartment (Occ. 8) and one neighbor (Occ. 18, below floor) is more exposed, respectively, +8% and +451%. In the high dilution scenario, Occ. 8 and Occ. 18 are also more exposed, respectively, +26% and +683%, also occupant 9 has an increase of 3% which is acceptable, compared with the benefits of the other occupants.
- In this geometry of apartment, opening one or more windows has no impact on occupant exposures. Indeed, scenarios 1, 4, and 5 give equivalent results. Only the scenario of half‐opening the windows gives slightly higher increases in exposure.

We are now refining the study with the second indicator, the probability of infection (Table 7). In the reference scenario, all occupants have less than a 1.6% probability of being infected by the virus, it is important that the proposed strategies do not increase this risk. We observe the same two groups of scenarios:

- All four scenarios of first group result in much higher risks of infection for all the occupants except Occ. 8, in particular Occ. 4 (5.4%, floor below), 9 (6.4%, same apartment), and 18 (3.65%, floor above), as well as Occ. 10 (reference apartment), with the highest probability of infection reaching almost 12% for the strategy of half‐opening the window. Nevertheless, this second indicator shows for the neighbor Occ.6 (0.7%, same floor), that despite an exposure increase of 65% the risk stays very low.
- The dilution strategies (sc 2&3) are much more effective as they allow almost all inhabitants to see their risk of infection decrease. Occupant 8 (reference apartment) is again an exception with a risk of infection considered as low (<1%).

Finally, the analysis of the inflows and outflows, cumulated over the simulation period, through the different walls of the quarantine room helps to understand these results (Table 8). In the reference case with the window closed, most of the air enters the quarantine room through the air inlet (trickle vent) and the leaks on the outside wall. Most of the air exits through the door undercut of the chamber. The other airflows are low and outgoing, except for airflow incoming from the floor (appt D). Concerning the studied scenarios, the results are again divided into two groups: the dilution strategies (sc.2&3), and the other strategies (sc. 1, 4, 5, and 6).

- In the scenarios of this second group, the airflows are almost identical. The only notable difference is in the semi‐open window scenario (sc. 6), where the airflow through the window is much lower. In all these scenarios, as the airflow through the door is null, the outgoing airflows are distributed to the other walls, with increasing values compared with the reference scenario: the airflow to the Appt. E (same floor, Occ. 6) is doubled, the one to the appt. U (above, Occ. 4) is multiplied by a 3‐factor. It should be noted that the airflow to the appt. D (below, Occ. 18) is inversed and doubled, explaining why the Occ. 18 is particularly impacted. Also, the air tends to disperse more toward the adjacent rooms, which is consistent with the increased probability of infection observed for the occupants located in adjacent rooms in the reference apartment and in the other apartments.
- For the dilution scenarios (2&3), the results of the air flows do not follow the same circuit. The airflow through the open door increases, and on the contrary, the airflows through the leaks of the other walls decrease, so the inhabitants of the neighboring dwellings see their probability of infection decrease. Logically, the inhabitants of the reference dwelling do not benefit from this decrease.

### 3.2 Sensitivity of the results to the two other ventilation systems

We calculate also these three indicators: relative exposures, probability of infection, and cumulated airflows through the walls of the quarantine room for the two other ventilation systems: the BV and RH‐DCV ones. We observe similar results on the following points:

- We observe the same groups of scenarios with different types of impacts.
- In the first group, the three occupants living in adjacent apartments experience high but lower increase in exposure, the lowest being obtained with the RH‐DCV: +38% (Occ. 6, same floor); +209% (Occ 4, floor above); +3265% (Occ 18, floor below). The probability of infection of the neighbor Occ. 4 (appt U, above) stays between 4% with BV and 6.4% with RH‐DCV. The probability of infection of the neighbor Occ. 6 (appt E, same floor) stays very low whatever the ventilation system (<1%)
- With the dilution strategies, the same tendency is observed with decreased exposures, but contrary to EV, all the occupants, including the neighbor Occ. 18 experience these decreases (−31; −94%). All occupants out of the apartments see their risk of infection decrease.
- Opening one or more windows has a very slight impact on occupant exposures and on probability of infection. It confirms that with this geometry of apartment, more the opening of the window, the sealing of the door has a strong negative impact on the occupants' exposure, whatever the ventilation system.
- The same tendencies are observed on the different airflows, and especially on the airflows to the other apartments. We observe the same inversion of airflow with Apart. D during the first group of strategies. Globally, these airflows are higher in absolute value than with the EV. For example, with the first group of scenarios, the airflow to the appt. D (below, Occ. 18) is inversed and multiplied by a 6‐factor with the BV and the RH‐DCV (only doubled with EV); the airflow to the appt. U (above, Occ. 4) is multiplied by a 4‐factor with the BV and by a 8‐factor with the RH‐DCV (only 3‐ with EV)

Only some differences to the EV system exist on the following points:

- For the BV and RH‐EV systems, all the scenarios are beneficial for the quarantined occupant (Occ. 11), with exposure decreases between −3 and −42%.
- In the reference cases, the probability of infection is lower with the BV (max. 1.15%), and higher with the RH‐DCV (max 2.04%), against 1.65% max with EV.
- The probability of infection of Occ. 18 (appt D, below) becomes of concern with RH‐DCV (5.98%, against 3.65% with EV and 3.5% with BV).

### 3.3 Discussion

In the residential building case study, opening the windows—including the window of the quarantine room—can decrease or increase the exposure of other occupants, including in neighbors' apartments. It will more depend on the position of the internal door of the quarantine room than on the ventilation system. This is mainly due to air leakage between adjacent rooms at increased flowrates and volumes due to the higher pressure differences caused by opening windows and sealing the door.

The observed overexposures result in increases in the risk of infection for the occupants. The comparison of infection risk values between different occupants makes it possible to determine which strategies favor or disadvantage the occupants who are already most at risk in the dwellings. In particular, they allow us to find the value of dilution strategies that would have been overlooked by the simple study of relative exposures. For example, in some cases, even if the exposure of some occupants is increased, a strategy becomes interesting because it lowers the maximum exposure risk of the most exposed occupant. In our case study of a multi‐family building, the estimation of the risk of infection shows the danger of opening the windows when the quarantine room is sealed, especially for occupants of neighboring dwellings who had a zero risk of being infected in the reference case.

In the light of these results, we can observe that this widespread recommendation, to open windows and to seal the door of a room where an infected person is quarantined, may be based on the consideration of a single zone model. This will nearly always decrease the exposure and probability of infection in the room where the windows are open (except in some cases highlighted in this study). In real multizone residential buildings, we have shown that this strategy should be considered with caution for occupants out of the zone where the infected people stays and especially out of the dwelling.

## 4 CONCLUSION AND PERSPECTIVES

Ventilation of buildings has been an area of intensive research for some time, due to the energy and health impacts associated with indoor air quality. In a virus pandemic context, ventilation of buildings has also been characterized as a risk in some cases (for instance, air recirculation), but overall offers a solution that could curb or even prevent aerosol transmission of a virus. As ventilation systems are responsible for pressure differences between zones in the same building, any recommendation for window opening and door sealing must take into account the airflows between all zones due to these pressure differences. In our simulations, considering one inhabitant in a quarantine room, we assess the risk of virus transmission for all inhabitants of a multi‐family dwellings due to these airflows in various scenarios. We observe that the recommendation to seal the door to the quarantine area, when a mechanical ventilation system is in operation, can strongly increase the probability of infection of several building occupants. These impacts can easily be explained by studying the airflows outgoing and entering the quarantine room, including the different leakages on the external and internal partitions, floors, and ceilings, and how they are impacted by the different strategies.

Nevertheless, as our simulations are for a single multi‐family dwelling, with many fixed values for parameters that can have a significant impact on the results, we are conducting further research to better understand the role of ventilation systems regarding airflows between zones.

We have illustrated the importance of moving from a single‐zone approach to a multizone approach to quantify the impacts of ventilation and airing in multizone buildings such as residences in order to prevent outbreaks of viruses such as SARS‐CoV‐2.

Before these results can be used to make general recommendations, they need to be studied in more detail. Firstly, sensitivity analyses need to be carried out for many parameters, such as air leakage level and distribution and pressure coefficient values. The need for databases with representative internal and external air leakage distributions from buildings worldwide is a crucial issue. Secondly, research needs to be conducted by experimenting with other climatic conditions (e.g., high wind areas), other building types, other types of ventilation systems, and other occupant scenarios. The impact of all these parameters should be quantified, with a characterization of the uncertainty of the result taking into account the uncertainty of the data from the on‐site measurements, the uncertainty due to the models and the representativeness of the values from the literature. In further studies, the transfer through the staircase will be an interesting contribution as it is not considered in this study. In the future, virus dilution strategies could be more promising, once the contaminating dose of the virus is known. In addition, these results need to be supplemented as soon as new information is published on the viability of the virus in the air, and on the characterization of aerosols that carry the virus.

## CONFLICT OF INTEREST

No conflicts of interest to declare.

## Data Availability

The data that support the findings of this study are available from the corresponding author upon reasonable request.
